# Multisport vs. Single-Sport Training and Motor Coordination in Children: A Quasi-Experimental Pre–Post Comparison

**DOI:** 10.3390/jfmk11030295

**Published:** 2026-07-27

**Authors:** Nicola Mancini, Rita Polito, Giovanni Messina, Marcellino Monda, Francesco Paolo Colecchia, Vlad Teodor Grosu, Simone Lombardi, Antonietta Messina, Siria Mancini, Fiorenzo Moscatelli

**Affiliations:** 1 Department of Education and Sport Sciences, Pegaso Telematic University, 80143 Naples, Italy; nicola.mancini@unipegaso.it; 2 Department of Psychology and Education, Pegaso Telematic University, 80143 Naples, Italy; rita.polito@unipegaso.it; 3 Section of Human Physiology and Unit of Dietetics and Sports Medicine, Department of Experimental Medicine, University of Campania “Luigi Vanvitelli”, 80138 Naples, Italy; giovanni.messina@unicampania.it (G.M.); marcellino.monda@unicampania.it (M.M.); francescocolecchia@gmail.com (F.P.C.); 4 Department of Mechatronics and Machine Dynamics, Universitatea Tehnica, 400114 Cluj-Napoca, Romania; vlad.grosu@mdm.utcluj.ro; 5 Department of Humanistic Studies, University of Foggia, 71121 Foggia, Italy; simonelombo94@gmail.com (S.L.); siria_mancini.585707@unifg.it (S.M.); 6 Department of Precision Medicine, University of Campania “Luigi Vanvitelli”, 80138 Naples, Italy; antonietta.messina@unicampania.it

**Keywords:** motor coordination, multisport training, children, KTK, motor development, early specialization

## Abstract

**Background:** This study aimed to compare changes in motor coordination associated with two different motor practice models—multisport and single-sport soccer training—in children, without implying causal effects of one training context over the other. **Methods:** A quasi-experimental pre–post study (T0–T1) was conducted over 10 weeks in non-randomized, pre-existing groups. A total of 143 children (mean age: 6.21 ± 0.55 years; 107 males) were allocated to a Multisport Group (MG; *n* = 79) and a Single-sport Group (SG; *n* = 64). Motor coordination was assessed using the Körperkoordinationstest für Kinder (KTK), and raw scores (RAW SCORE) were used for analysis. Statistical analyses included *t*-tests, change-score (Δ) and effect-size analyses, and two-way repeated-measures ANOVA. **Results:** Both groups showed significant improvements over time (*p* < 0.001; d = 0.75 for MG, d = 0.77 for SG). However, the MG showed significantly greater improvements than the SG (Δ = 16.68 ± 6.14 vs. 12.28 ± 4.08; *p* < 0.001; d = 0.83). At T1, the SG still showed slightly higher absolute RAW SCORE values than the MG (*p* = 0.049; d = −0.33), although this between-group difference was markedly reduced compared to baseline (d = −0.59). The ANOVA revealed a very large effect of Time (F(1,141) = 1092.31, *p* < 0.001, η^2^p = 0.886), a small-to-medium effect of Group (F(1,141) = 7.51, *p* = 0.007, η^2^p = 0.051), and a large Time × Group interaction (F(1,141) = 24.18, *p* < 0.001, η^2^p = 0.146). A sensitivity analysis (ANCOVA) confirmed that, after adjusting for baseline scores, sex, and anthropometric variables, the Multisport group showed significantly higher adjusted T1 scores than the Single-sport group (adjusted mean difference = 3.96 points, 95% CI: 1.93–5.99), reversing the direction observed in the unadjusted T1 comparison. Given the non-randomized design and baseline group differences, findings reflect observed associations rather than causal effects. **Conclusions:** Both practice models were associated with improved motor coordination, with multisport participation associated with greater observed change over 10 weeks. These findings are consistent with a possible role of diversified motor experiences in coordination development, though this study cannot establish causality.

## 1. Introduction

Motor coordination is a fundamental component of motor competence and plays a key role in children’s physical and psychosocial development, and has been linked in the literature to physical activity and long-term health trajectories [1,2,3,4]. Higher coordination levels are associated with greater physical activity participation [5,6] and healthier developmental trajectories, whereas low coordination has been linked to reduced activity levels and increased risk of adverse health outcomes, including excess body weight [1,4,7,8]. These associations, established primarily through longitudinal and cross-sectional research, provide the broader rationale for studying coordination; the present study, however, focuses specifically on short-term (10-week) KTK performance changes and does not assess downstream health outcomes. In the Italian context, recent evidence has raised concerns about declining motor opportunities in children, consistent with international evidence showing a decline in motor competence levels across recent decades, likely associated with reduced exposure to diverse and enriched movement experiences [4,7,9,10,11]. Early childhood represents a sensitive period for neuromotor development, during which not only the quantity but also the quality and variability of practice appear crucial, as diversified motor experiences are associated with higher coordination levels and broader motor repertoires [1,12,13]. The early diversification model suggests that children exposed to a wide range of motor experiences develop a broader and more adaptable motor repertoire, with potential benefits for long-term motor competence and reduced injury risk, whereas early single-sport specialization has been associated with reduced movement variability and negative outcomes such as overuse injuries [13,14]. It should be noted that this evidence base predominantly derives from longitudinal or multi-year observational studies; whether comparable benefits emerge over a short-term, 10-week training window, as examined in the present study, has not been directly established and remains an empirical question this study aims to address. These concepts align with the ecological dynamics framework, which describes motor behavior as emerging from the interaction among the individual, the task, and the environment, with variability acting as a functional property that supports exploration, adaptation, and flexible, context-sensitive movement solutions [15,16]. Accordingly, multisport programs—characterized by diverse tasks and non-specialized practice—may enhance motor adaptability and transferable coordination skills more effectively than early single-sport training. Children engaged in multiple sports generally demonstrate higher levels of gross motor coordination compared to those specializing early [12,13,17], and Popović et al. [8] reported significantly higher motor coordination in children engaged in multisport activities compared to soccer-only training. In the Italian context, Mancini et al. [9] similarly found that structured multisport programs are associated with higher coordination levels than traditional physical education. However, a large proportion of the available literature is based on cross-sectional or observational designs, limiting causal inference, while controlled studies examining the effects of different training models in early childhood remain less represented [4,8,18]. Building on this earlier cross-sectional evidence, including our own prior work on multisport participation and coordination [9], the present study extends this line of research by adopting a pre–post design in pre-existing training groups, allowing within-subject change over a defined 10-week period to be directly quantified—an aspect that cross-sectional comparisons cannot address. The assessment of motor coordination is commonly performed using the Körperkoordinationstest für Kinder (KTK), a widely adopted, non-sport-specific tool for children aged 5–14 years that demonstrates good reliability and sensitivity in detecting differences related to physical activity levels and sport participation [4,12,18]. Although the KTK shows good reliability and sensitivity to training-related differences, its interpretation requires caution due to the use of historical normative values; in this context, raw scores may provide a more sensitive measure of within-subject changes over time compared to normalized indices [7], particularly when the aim is to capture intervention-related adaptations. To the best of our knowledge, this is among the first quasi-experimental pre–post studies conducted under real-world training conditions to directly compare multisport and single-sport soccer practice in children aged 5–7 years, while combining complementary statistical approaches, including repeated-measures ANOVA, ANCOVA, and mixed-effects models, to evaluate changes in motor coordination over time. Therefore, the aim of the present study was to compare motor coordination development associated with multisport and single-sport soccer training in children aged 5–7 years using a quasi-experimental pre–post design. It was hypothesized that both training models would be associated with improvements in motor coordination (assessed via KTK raw scores), but that the multisport approach would be associated with greater improvements, consistent with greater diversity of motor experiences typically characterizing multisport programs, although this diversity and its underlying mechanisms were not directly measured in the present study.

## 2. Materials and Methods

### 2.1. Study Design

This study employed a quasi-experimental pre–post design with non-randomized, pre-existing groups over a 10-week period. The aim was to compare motor coordination development associated with two different motor practice models (multisport vs. single-sport soccer training) in children. Participants were already engaged in structured training programs that began at the start of the sports season (September 2024); prior training exposure at the time of enrollment was approximately 4 months for all participants, quantified as time elapsed since program start, and did not differ significantly between groups. This pre-existing exposure may have introduced selection bias due to group characteristics that predate the study. To minimize the influence of early adaptation effects and transient changes associated with the initial training phase, data collection was conducted during the mid-season period, defined as weeks 14–24 of the approximately 8-month season. A 10-week observation window was selected to ensure stable training conditions. Assessments were performed at two time points: T0 (baseline) and T1 (post-test, after 10 weeks). All participants completed both assessments, and no dropouts were recorded.

### 2.2. Participants

A total of 143 children participated in the study (mean age: 6.21 ± 0.55 years), including 107 males and 36 females. Participants were assigned to groups based on their pre-existing training background rather than individual randomization. Because allocation reflected family- and club-level choices made prior to the study, unmeasured factors such as parental sport involvement, socioeconomic context, and prior motor experience may have differed systematically between the Multisport and Single-sport groups. The Multisport Group (MG; *n* = 79; 55 males, 24 females) consisted of children who had been regularly involved for at least one year in a structured multisport program, characterized by exposure to multiple sports and diversified motor activities. The Single-sport Group (SG; *n* = 64; 52 males, 12 females) included children engaged exclusively in structured soccer training for at least one year, with no participation in other organized sports activities. The selected age range (5–7 years) corresponds to a sensitive developmental period for motor coordination. Participants were recruited from multiple local sports clubs (ASD) already offering either multisport programs or soccer-only training, with children sampled within each club without preference criteria. Since group allocation reflected the type of program offered by each club rather than individual randomization, the design remains quasi-experimental. Potential baseline differences between groups were examined through between-group comparisons, ANCOVA, and the inclusion of interaction effects in the repeated measures ANOVA; these procedures adjust for the measured covariates but cannot fully remove the influence of unmeasured confounding inherent to a non-randomized design. The use of real-world, pre-existing training groups enhances the ecological validity of the study, reflecting typical practice conditions in youth sport settings, although pre-existing disparities in motor competence between groups cannot be fully excluded and should be considered when interpreting the results. The study was conducted in accordance with the Declaration of Helsinki. Written informed consent was obtained from all parents or legal guardians; in addition, verbal assent was obtained from each child using age-appropriate explanations prior to testing, and any expressed reluctance would have led to exclusion from data collection. The study protocol was approved by the Ethics Committee of the Pegaso Telematic University (PROT/E 002466, 29 March 2024).

### 2.3. Measurements

#### 2.3.1. Anthropometric Characteristics

Height (in cm) and weight (in kg) were measured using standardized instruments (stadiometer and electronic scale). The measurements were taken concurrently with the administration of the KTK test, at the beginning of the evaluation period. Body mass index (BMI) was calculated as weight/height^2^ (kg/m^2^) and classified according to age- and sex-specific percentiles [19] (Table 1).

#### 2.3.2. Inclusion and Exclusion Criteria

Inclusion criteria were: continuous participation (≥1 year) in either the assigned multisport program or soccer-only training; a minimum frequency of two training sessions per week (≥60 min/session); no participation in organized sports activities outside the assigned program (i.e., Multisport Group children were enrolled exclusively in their multisport club program, with no additional single-sport training; Single-sport Group children were enrolled exclusively in soccer, with no participation in other sports); and attendance ≥ 80% of scheduled sessions. Mean attendance over the 10-week observation period was 91% in the Multisport Group and 87% in the Single-sport Group, indicating adequate adherence in both groups. Exclusion criteria were: certified disabilities, recent injuries, incomplete data, or attendance below 80%.

#### 2.3.3. Motor Coordination—KTK Test

Motor coordination was assessed using the Körperkoordinationstest für Kinder (KTK), a validated and reliable tool for assessing gross motor coordination in children aged 5–14 years, originally developed by Kiphard and Schilling [20], and widely adopted as a standardized assessment of gross motor coordination in children. The KTK has been widely used in both cross-sectional and longitudinal studies as a non-sport-specific measure of gross motor coordination and demonstrates good reliability and sensitivity in detecting differences related to physical activity and sport participation [4,12,18]. Previous studies have reported high test–retest reliability for the KTK, with intraclass correlation coefficients (ICC) typically ranging from moderate to excellent reliability across subtests [18]. In addition, the test shows good construct validity, as KTK performance has been consistently associated with physical activity levels, physical fitness, and motor competence indicators in children [4,12]. From a theoretical perspective, the multidimensional structure of the KTK aligns with ecological dynamics principles, as the subtests require continuous adaptation to task and environmental constraints, thereby capturing functional aspects of motor coordination and adaptability. However, caution is warranted when interpreting standardized scores, as normative values are based on historical reference samples and may not fully reflect contemporary populations. The test includes four subtests: Walking Backward (WB, dynamic balance), Hopping for Height (HH, unilateral coordination and power), Jumping Sideways (JS, speed and coordination), and Moving Sideways (MS, global coordination and rhythm). All assessments were administered by trained evaluators with degrees in Sports and Movement Sciences and prior experience with the KTK protocol. Standardization sessions were conducted before data collection to minimize inter-rater variability; however, formal inter-rater reliability indices (e.g., intraclass correlation coefficients) were not calculated for this specific sample, and this is acknowledged as a limitation. For statistical analysis, raw scores (RAW SCORE) were used to directly capture changes in motor performance and better reflect within-subject variations over time, avoiding potential distortions related to age-based normalization procedures. This approach is consistent with previous literature highlighting the sensitivity of raw scores to developmental changes and their suitability for detecting performance variations compared to normalized indices such as the Motor Quotient (MQ) [7]. The total score (RAW SCORE) was calculated as the sum of the four subtests. To improve comparability with prior studies reporting standardized outcomes, age- and sex-adjusted Motor Quotient values were not computed in the present analysis; this is acknowledged as a limitation, as MQ-based sensitivity analyses would allow more direct comparison with normative-based literature.

### 2.4. Training Programs

Participants were involved in structured training programs already implemented within their respective sports clubs. During the 10-week observation period, training volume was comparable between groups (2 sessions/week, 60 min each; approximately 120 min/week). To control for potential differences in internal load, perceived exertion was assessed using the RPE-C scale [21], a pictorial scale specifically developed and validated for young children who do not read, featuring seven images showing a man becoming progressively fatigued. Prior to the first session, a standardized familiarization procedure was conducted, in which each child was guided through the scale using concrete examples calibrated to their experience (e.g., ‘how do you feel when you run very fast?’ for high exertion; ‘how do you feel when you walk slowly?’ for low exertion). The scale was administered individually at the end of every training session, within 1–2 min of its conclusion, by an external evaluator; each child was approached separately from the group to prevent social influence on responses. Mean RPE-C values, rated on the 7-point pictorial scale, were descriptively similar between groups (MG: 7.6 ± 0.3; SG: 7.8 ± 0.2). However, RPE-C reflects perceived exertion only and does not capture training volume, density, neuromotor load, or task complexity; therefore, comparable RPE values do not fully rule out differences in internal training load between programs. It should be acknowledged that the validity of RPE self-report in children aged 5–7 years remains debated; however, the RPE-C was specifically developed and validated for children aged 5–6 years who do not read [21], and the use of individual administration and prior familiarization represent methodological precautions consistent with current recommendations for this age group.

The Multisport program was characterized by greater diversity of motor experiences and a multilateral approach aimed at promoting global motor development, involving a broader range of task types compared to the Single-sport program; motor variability itself was not directly quantified and is inferred from program design rather than measured. Each session included two 15 min game-based activities specifically designed to maximize movement variability, combined with balance exercises, ball activities targeting hand–eye coordination, and obstacle-based motor circuits, targeting stability, locomotor, and manipulative skills with gradual progression. Example tasks included balance-beam crossing progressing from wide to narrow supports, and two-handed ball catching at increasing distances. The Single-sport program was based on structured soccer training, including technical drills (ball control, passing, shooting), coordination exercises with the ball, and small-sided games (e.g., 3 vs. 3, 4 vs. 4), with progression from simplified drills to game-based scenarios. The main characteristics of the two programs are summarized in Table 2.

### 2.5. Statistical Analysis

Statistical analyses were performed using IBM SPSS Statistics (Version 25.0, IBM Corp., Armonk, NY, USA). Data are presented as mean ± standard deviation (SD). Normality of distribution was assessed using the Shapiro–Wilk test. Visual inspection of boxplots and histograms was also performed to detect potential outliers. All analyses were conducted using RAW SCORE from the KTK test to better capture within-subject performance changes over time and avoid age-related normalization effects. The following analyses were conducted: paired *t*-tests for within-group comparisons (T0 vs. T1); independent *t*-tests for between-group comparisons; analysis of change scores (Δ = T1 − T0); and calculation of effect sizes (Cohen’s d). Additionally, a two-way repeated measures ANOVA was performed, with Time (T0 vs. T1) as the within-subject factor and Group (Multisport vs. Single-sport) as the between-subject factor, to assess main effects and interaction. Because participants were nested within five clubs per group, a linear mixed-effects model was additionally fitted on RAW SCORE, with Time and Group as fixed effects and random intercepts for club and for participants nested within club, to account for potential club-level clustering. Effect sizes (Cohen’s d) were interpreted according to conventional thresholds: <0.20 trivial, 0.20–0.49 small, 0.50–0.79 moderate, and ≥0.80 large. For ANOVA, partial eta squared (η^2^p) was calculated as a measure of effect size and interpreted according to conventional thresholds for this statistic (≈0.01 small, ≈0.06 medium, ≈0.14 large), distinct from the Cohen’s d thresholds used for pairwise comparisons. All tests were two-tailed, with significance set at *p* < 0.05. To account for the multiple comparisons performed across the four KTK subtests and the total RAW SCORE (within-group, between-group, and change-score comparisons; 25 tests in total), a Benjamini–Hochberg false discovery rate (FDR) correction was additionally applied; corrected q-values for all 25 comparisons are reported in Table S1 (Supplementary Materials). As a sensitivity analysis, an analysis of covariance (ANCOVA) was performed on T1 scores using T0 scores as a covariate, in order to verify that the observed group differences were not solely attributable to baseline differences (i.e., regression toward the mean). Given the unequal sex distribution between groups, a second ANCOVA model additionally including sex as a covariate was performed.

### 2.6. Statistical Power Analysis

An a priori power analysis was conducted using G*Power 3.1 to determine whether the available sample size was adequate to detect meaningful between-group differences. Assuming a medium effect size (Cohen’s d = 0.50), a significance level of α = 0.05, and a desired statistical power of 0.80, the minimum required sample size was estimated at 128 participants (64 per group). The final sample consisted of 143 children (Multisport Group: *n* = 79; Single-sport Group: *n* = 64), exceeding the required sample size, indicating that the study was adequately powered to detect meaningful differences between groups and reduce the risk of Type II error. The power analysis assumed independent observations and did not account for clustering by sports club. Using the intraclass correlation from the club-level model (Section 3.9, ICC = 0.21) and an average cluster size of 13–16 children, the design effect was approximately 3.5–4.1, corresponding to an effective sample size of approximately 18–19 participants per group. Statistical power for subtest-level and exploratory comparisons is likely lower than for the primary outcome; the FDR correction (Section 2.5) was applied to control the overall error rate across these comparisons.

## 3. Results

### 3.1. Preliminary Analyses

Normality of raw variables was verified using the Shapiro–Wilk test and visual inspection of the distributions, with no extreme outliers detected. Residuals from the ANCOVA model (Section 3.8) departed from normality (Shapiro–Wilk W = 0.844, *p* < 0.001). The model was therefore re-estimated using heteroscedasticity-consistent (HC3) robust standard errors, confirming the significance of the group effect (β = 4.21, *p* < 0.001, 95% CI: 2.05–6.37).

### 3.2. Descriptive Statistics of KTK Subtests and Total Score

Descriptive statistics for the KTK subtests and total score are reported in Table 3. In both groups, an average increase between T0 and T1 was observed across all parameters. In the Multisport group, the most marked improvements concerned Walking Backward (WB), Jumping Sideways (JS), and the total score. In the Single-sport group, a more homogeneous increase was observed, with substantial gains particularly in the total score and in the subtests related to soccer-specific coordination.

### 3.3. Within-Group Comparisons

Within-group comparisons revealed statistically significant improvements between T0 and T1 across all subtests and the total score, in both groups (Table 4). For the total score (RAW SCORE), the increase was significant in both groups, with a large effect size in both cases (Multisport: *p* < 0.001, d = 0.75; Single-sport: *p* < 0.001, d = 0.77). At the subtest level, in the Multisport group the largest effects were observed for Jumping Sideways (JS; d = 0.61) and Walking Backward (WB; d = 0.58), whereas in the Single-sport group the largest effect was observed for Moving Sideways (MS; d = 0.81), followed by Hopping for Height (HH; d = 0.62).

### 3.4. Between-Group Comparisons

At baseline (T0), the Single-sport group showed significantly higher values than the Multisport group for the total score (*p* < 0.001; d = −0.59), as well as for the WB and HH subtests (Table 5). Because groups differed at baseline, between-group comparisons at T1 and change-score analyses (Section 3.5) are interpreted alongside the ANCOVA and mixed-effects models (Section 3.8 and Section 3.9), which adjust for baseline differences and club-level clustering. At post-test (T1), the initial advantage of the Single-sport group was reduced but not completely eliminated. For the total score, the Single-sport group maintained significantly higher values (*p* = 0.049; d = −0.33), although with a markedly smaller effect size compared to baseline (from d = −0.59 to d = −0.33). At the subtest level, significant differences in favor of the Single-sport group persisted for WB (*p* < 0.001; d = −0.66) and HH (*p* = 0.005; d = −0.48), whereas no significant difference emerged for MS and JS, either at T0 or at T1 (Table 5). These results indicate a substantial, although not complete, reduction in the initial gap between groups over the observation period. Despite the initial advantage of the Single-sport group, the Multisport group showed a marked reduction in the gap over time, consistent with the greater increase observed in the analysis of change scores (Section 3.5).

### 3.5. Analysis of Change Scores (ΔT1–T0)

**Figure 1:**
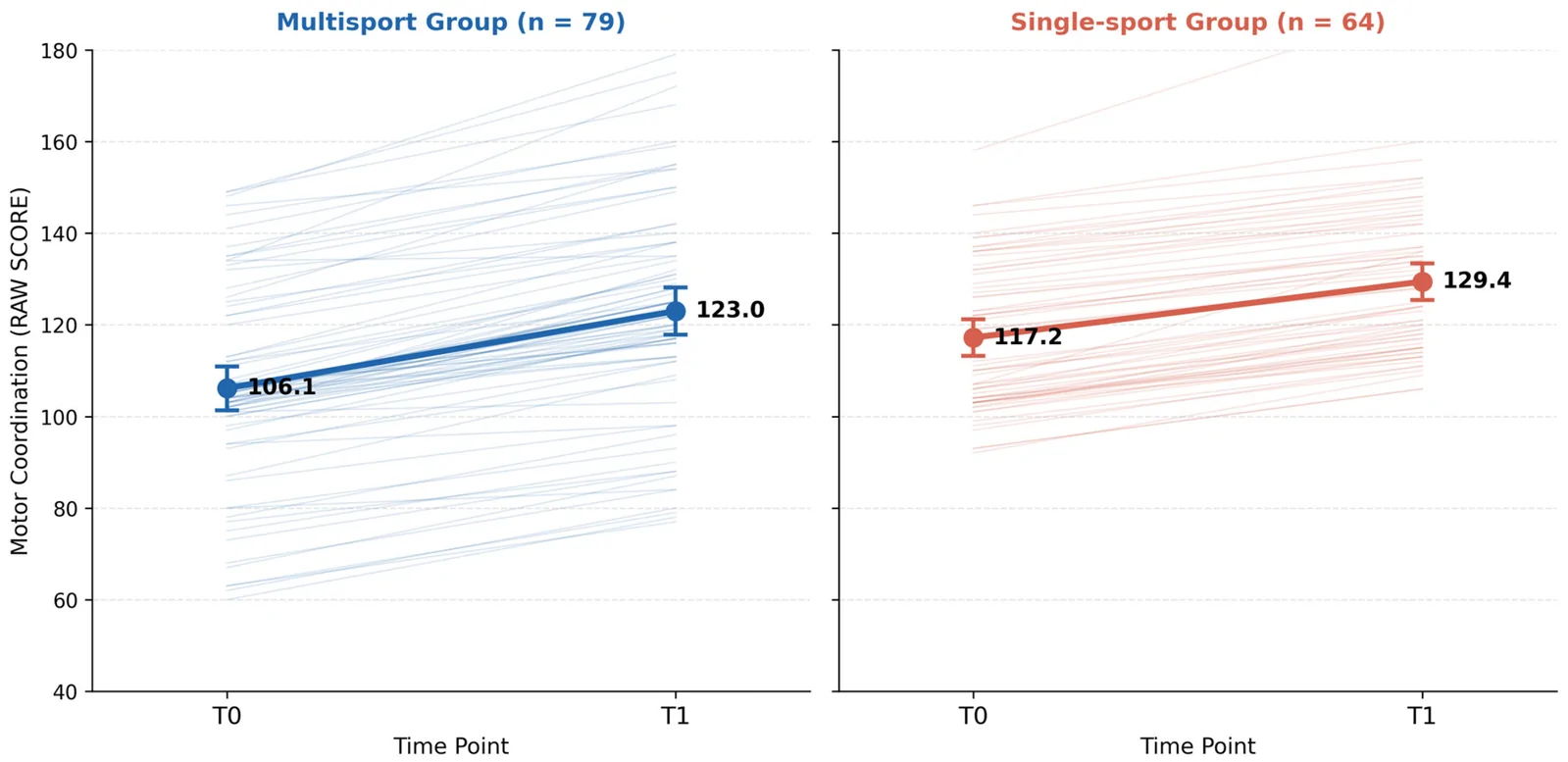
Individual trajectories of motor coordination (RAW SCORE) from T0 to T1 in the Multisport Group (**left**) and Single-sport Group (**right**). Thin lines represent individual participants. Circles represent group means; error bars indicate 95% confidence intervals. Both groups showed significant within-group improvements (*p* < 0.001).

**Figure 2:**
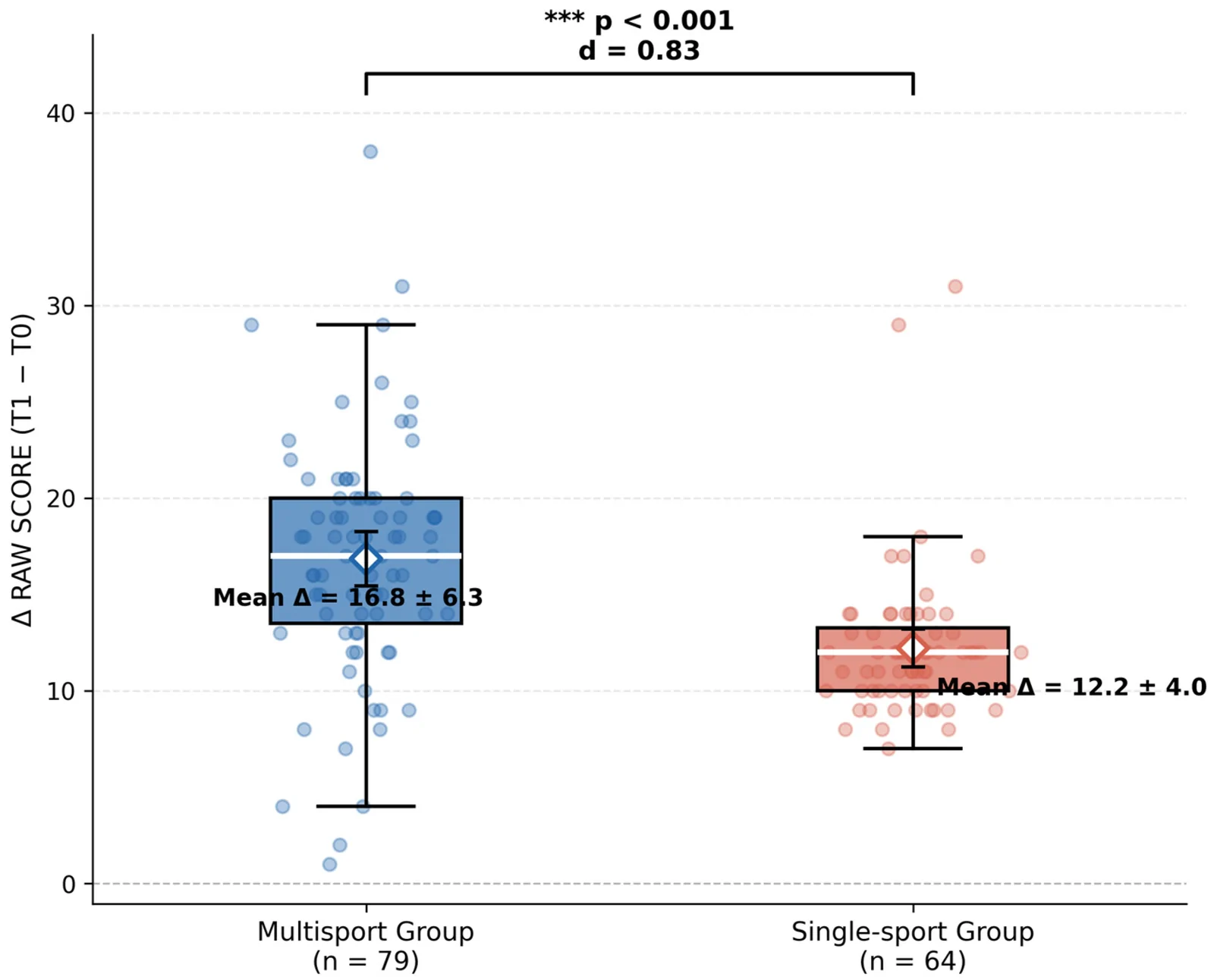
Distribution of individual change scores (Δ RAW SCORE = T1 − T0) by group. Dots represent individual participants (jittered for clarity). Box plots display median and interquartile range; whiskers extend to 1.5 × IQR. Diamonds represent group means ± 95% CI. *** *p* < 0.001; Cohen’s d = 0.83.

The analysis of change scores is reported as a secondary, descriptive indicator alongside the ANCOVA and mixed-effects estimates (Section 3.8 and Section 3.9), which represent the primary between-group comparisons given the baseline differences noted in Section 3.4. The Multisport group showed a greater increase than the Single-sport group in raw change scores (Multisport: +16.68 ± 6.14; Single-sport: +12.28 ± 4.08; *p* < 0.001; d = 0.83), consistent in direction and magnitude with the covariate-adjusted estimates. At the subtest level, the increase was significantly greater in the Multisport group for WB (*p* < 0.001; d = 0.99) and JS (*p* < 0.001; d = 0.87), whereas no significant differences emerged for MS (*p* = 0.940) and HH (*p* = 0.230). A two-way repeated measures ANOVA was also performed, with Time (T0 vs. T1) as the within-subject factor and Group (Multisport vs. Single-sport) as the between-subject factor, in order to assess the main effects and their interaction (Table 6). The analysis revealed a significant effect of Time (*p* < 0.001), a significant effect of Group (*p* = 0.007), and a significant Time × Group interaction (*p* < 0.001), indicating a significantly greater improvement in the Multisport group compared to the Single-sport group over time (Table 7). According to conventional thresholds for η^2^p (small ≈ 0.01, medium ≈ 0.06, large ≈ 0.14), the effect of Time was very large (η^2^p = 0.886), the effect of Group was small-to-medium (η^2^p = 0.051), and the Time × Group interaction was large (η^2^p = 0.146). These results are consistent with the analysis of change scores and with the graphical representations reported in Figure 1 and Figure 2.

### 3.6. Graphical Analysis

The time course of the total motor coordination score (RAW SCORE) is reported in Figure 1. Both groups showed a significant increase between T0 and T1 (*p* < 0.001), confirming the effectiveness of the two practice models in improving motor coordination over the observation period. The analysis of change scores (ΔT1–T0), reported in Figure 2, showed a significantly greater increase in the Multisport group compared to the Single-sport group (*p* < 0.001; Cohen’s d = 0.83), indicating an advantage of the multisport model in terms of overall improvement in coordination.

### 3.7. Summary of Results

Overall, both programs produced significant improvements in motor coordination over the observation period. However, despite the Single-sport group showing significantly higher initial levels, the Multisport group showed significantly greater increases over time. The analysis of change scores and the significant Time × Group interaction consistently indicate a greater coordinative adaptation associated with the multisport model.

### 3.8. Sensitivity Analysis: Adjustment for Baseline Differences and Sex

Because the Single-sport group showed significantly higher RAW SCORE values at baseline, an ANCOVA was performed on T1 RAW SCORE with T0 RAW SCORE as a covariate (Group coded as Single-sport = reference, Multisport = comparison), in order to verify that the observed group effect on T1 scores was not solely attributable to regression toward the mean. The homogeneity-of-slopes assumption was confirmed by a non-significant T0 × Group interaction (*p* = 0.117). After adjustment for baseline, the Multisport group showed significantly higher adjusted T1 scores than the Single-sport group (adjusted mean difference = 4.95 points, 95% CI: 3.08–6.81, *p* < 0.001). This adjusted result is directionally opposite to the unadjusted T1 comparison (Section 3.4), where the Single-sport group scored higher in absolute terms; the reversal reflects the fact that the unadjusted comparison reflects final performance level, whereas the adjusted comparison reflects the magnitude of change relative to each group’s own baseline.

Subtest-level ANCOVAs confirmed significant adjusted group effects for WB (*p* = 0.001) and JS (*p* < 0.001), but not for HH (*p* = 0.223) or MS (*p* = 0.829). Given the unequal sex distribution between groups (MG: 55 males/24 females; SG: 52 males/12 females), a second ANCOVA model additionally including sex as a covariate was performed. Sex was not a significant predictor of T1 RAW SCORE in this model (β = −1.11, *p* = 0.289), and the adjusted group effect remained materially unchanged (β = 4.81, 95% CI: 2.93–6.69, *p* < 0.001), indicating that the observed group differences were not attributable to the sex imbalance between groups. Given the developmental relevance of age in a 5–7-year-old sample, age was included as an additional covariate in a further ANCOVA model alongside T0 RAW SCORE, sex, height, and body mass. Age was not a significant predictor of T1 RAW SCORE (β = −1.24, *p* = 0.362), and the group effect remained significant after this adjustment (β = 4.50, *p* < 0.001, 95% CI: 2.05–6.94). Finally, the application of a Benjamini–Hochberg FDR correction across the 25 statistical comparisons reported in Table 4, Table 5 and Table 6 did not change the pattern of significant results: all comparisons significant at *p* < 0.05 in the uncorrected analysis remained significant after correction (all q < 0.05), and no previously non-significant comparison became significant. As a further sensitivity check, an additional ANCOVA model was fitted including T0 RAW SCORE, sex, body height, and body mass simultaneously as covariates, to address potential confounding from the anthropometric differences observed at baseline. Neither height (entered in meters; β = 21.32, *p* = 0.256), body mass (entered in kilograms; β = −0.09, *p* = 0.853), nor sex (β = −1.00, *p* = 0.344) were significant predictors of T1 RAW SCORE; the height coefficient reflects the effect of a 1 m increase and corresponds to approximately 6–9 points across the observed height range in this sample (0.3–0.4 m). The group effect remained highly significant (β = 3.96, 95% CI: 1.93–5.99, *p* = 0.0002), with adjusted means of 127.62 for the Multisport group and 123.67 for the Single-sport group. These results confirm that the observed group differences in motor coordination at T1 are not attributable to baseline anthropometric differences between groups.

### 3.9. Sensitivity Analysis: Club-Level Clustering

To account for potential clustering at the club level, a linear mixed-effects model was fitted with random intercepts for club and for participants nested within club. The Time × Group interaction remained significant (β = 4.93, SE = 1.07, *p* < 0.001, 95% CI: 2.84–7.02), confirming that the greater improvement observed in the Multisport group was robust to club-level clustering.

## 4. Discussion

The present study examined changes associated with two different motor practice models—multisport and single-sport soccer training—on motor coordination development in early childhood, using a quasi-experimental design with pre-existing groups and pre–post measurements during a stable phase of the sports season. The main finding shows that both programs produced significant improvements in coordinative abilities. However, the adaptation pattern differed between groups: the Single-sport group presented higher initial levels, while the Multisport group showed significantly greater increases over time. This result is supported both by the analysis of change scores and by the significant Time × Group interaction emerging from the repeated measures ANOVA.

### 4.1. Interpretation of Main Findings

The improvement observed in both groups is consistent with the hypothesis that systematic exposure to motor stimuli plays an important role in coordinative development during childhood. This finding is consistent with longitudinal evidence showing that participation in physical activity and sport is associated with significant increases in motor competence over time [4,12,22]. Moreover, these results fit within the theoretical framework describing motor competence as a dynamic and bidirectional construct, continuously related to physical activity levels and to health development trajectories [1,23]. From this perspective, the observed improvement does not represent a simple training effect, but the outcome of a progressive interaction between practice, neuromotor development, and functional adaptations. It is worth noting that these effects emerge under ecologically valid conditions, since participants were embedded in real training contexts, increasing the transferability of the results to youth sport practice. However, the larger changes observed in the Multisport group are consistent with the possibility that coordinative development is related not only to the amount of practice, but also to the quality, variability, and structure of the stimuli provided; this interpretation is inferred from the program descriptions in Section 2.4 and was not directly measured in the present study. The more marked increases in the total score (RAW SCORE) and in the Walking Backward (WB) and Jumping Sideways (JS) subtests suggest an improvement in dynamic balance, postural control, and intersegmental coordination, abilities that are strongly sensitive to variable and non-linear learning contexts. In the literature, these components are considered key indicators of motor system adaptability and of the capacity to manage multiple constraints [15,24]. In parallel, the developmental model proposed by Côté and colleagues highlights how the “sampling” phase, or early diversification, represents a crucial step for long-term development, as it favors not only more versatile motor skills but also greater motivation and adherence to sport practice [25,26]. This is further supported by evidence linking motor competence to indicators of physical fitness, body composition, and general health, highlighting how a rich and varied motor environment can generate lasting benefits [27,28,29,30,31]. Finally, the results of the present study are consistent with the concept of early diversification, according to which exposure to multiple motor experiences in early childhood contributes to the development of a more flexible, efficient, and resilient motor system [13,14]. Within this framework, overall motor coordination plays a central role not only as a learning outcome, but also as a prerequisite for active participation in physical activity and for maintaining positive health trajectories across the lifespan [22,23].

### 4.2. Role of Motor Variability and the Multisport Approach

From a theoretical perspective, the results can be interpreted in light of ecological dynamics and non-linear motor learning models, according to which motor behavior emerges from the interaction between the individual, the task, and the environment [15,16,24,32]. The multisport program, characterized by greater task diversity, may have offered richer opportunities for motor exploration and adaptable movement strategies [15,33,34,35], consistent with evidence linking early diversification to more robust motor competencies [13,25]. The single-sport model, relying predominantly on repetitive, discipline-specific drills, may have offered comparatively fewer such opportunities. The higher initial (T0) values observed in the Single-sport group likely reflect prior soccer-specific training history rather than an effect occurring within the observation window; the sensitivity analysis (Section 3.8) confirmed that the group difference in Δ RAW SCORE remained significant after adjusting for T0 scores. This pattern is broadly consistent with longitudinal evidence linking diversified motor experience to more favorable coordination and physical activity trajectories [1,4,12,13,22,23,36,37,38].

Although the findings are interpreted within the ecological dynamics framework, variables such as movement variability and affordances were not directly measured. Therefore, these interpretations remain theoretical and should be confirmed by future studies.

### 4.3. Significance of the Time × Group Interaction

The significant Time × Group interaction represents the most relevant inferential finding of the study, indicating differences in adaptation patterns between groups over time: the two groups did not improve in the same way over time, and the Multisport group was associated with a greater magnitude of change over time. This pattern is consistent with, though not a direct test of, theoretical accounts linking practice variability to motor exploration [24]. In applied terms, these results suggest that stimulus variability does not only influence the magnitude of improvement, but also the temporal dynamics of coordinative adaptation. It is worth noting that, at T1, the Single-sport group maintains a statistically significant advantage in the total score (*p* = 0.049; d = −0.33), although this advantage is markedly reduced compared to baseline (d = −0.59). At the same time, the analysis of change scores (Δ) shows a significantly greater increase in the Multisport group (d = 0.83). The combination of these two findings indicates that the Multisport group is progressively closing the initial gap, although it had not yet fully closed it within the 10 weeks observed. This pattern is particularly relevant in studies involving childhood, where adaptations may require longer periods to fully manifest. It is also worth noting that, although JS showed one of the largest relative improvements (Δ d = 0.87), this increase did not translate into a statistically significant between-group difference at T1 (*p* = 0.083). This can be explained by the fact that the JS subtest shows greater overall variability at T1 (pooled SD ≈ 7.4) compared to WB (pooled SD ≈ 6.1): the same absolute gain, when expressed against a larger denominator, produces a smaller standardized between-group difference at a single time point, even when the within-subject change is itself large. Consistently, the analysis of effect sizes—interpreted according to conventional thresholds for η^2^p (small ≈ 0.01, medium ≈ 0.06, large ≈ 0.14)—showed a very large effect for the Time factor (η^2^p = 0.886), a small-to-medium effect for Group (η^2^p = 0.051), and a large effect for the Time × Group interaction (η^2^p = 0.146). The large effect size of the Time × Group interaction confirms a meaningful difference in adaptation patterns between the two training models.

### 4.4. Functional Interpretation of the Subtests (Secondary, Exploratory Analysis)

The analysis of the individual KTK subtests is exploratory and secondary to the primary total RAW SCORE outcome; it provides further interpretive elements but was not the basis for the a priori power calculation (Section 2.6). In the Multisport group, greater improvements are observed in WB (dynamic balance) and JS (spatio-temporal coordination and speed), abilities that require continuous adaptation, high motor flexibility, and the integration of multiple movement components, and which are recognized in the literature as components highly sensitive to constraint variability and to the quality of motor experience [15,18,24]. In particular, dynamic balance and rhythmic-temporal coordination represent key indicators of the motor system’s adaptive capacity and of the ability to manage complex tasks in variable contexts [4,39]. In contrast, in the MS and HH subtests, the improvements are similar between groups, suggesting that some more structured components, or those more closely related to neuromuscular and strength factors, may be less sensitive to stimulus variability and more dependent on specific adaptations [28,29,40]. Overall, these exploratory subtest-level patterns are consistent with the possibility that multisport practice favors more adaptive and transferable coordinative components, though this should be confirmed by studies specifically powered for subtest-level comparisons [1,4,22,23,38]. It should be noted that the a priori power analysis was conducted on the primary outcome (total RAW SCORE); statistical power for subtest-level comparisons may therefore be lower, and findings at this level should be interpreted with appropriate caution, particularly for subtests showing smaller or non-significant between-group differences such as MS and HH.

### 4.5. Comparison with the Literature

Comparative evidence further supports the advantage of multisport contexts over mono-disciplinary practice. In particular, Popović et al. [8] reported significantly higher levels of motor coordination in children involved in multisport activities compared to soccer, with relevant differences across KTK subtests. In the Italian context, recent evidence confirms an advantage in coordination scores in children involved in multisport programs [9], reinforcing the role of stimulus variability as a determining factor in coordinative development. It should nevertheless be considered that a large part of the literature is based on observational designs, limiting causal inference [22]. In this sense, the present study, through a pre–post design under ecologically valid conditions, provides preliminary support for the hypothesis that exposure to variable motor stimuli is associated with greater improvements in coordination, although the lack of randomization and the presence of baseline differences between groups prevent firm causal conclusions. These results are particularly relevant in light of the evidence indicating a progressive decline in motor competence in youth populations, associated with a reduction in movement opportunities [41,42], and reinforce the importance of multisport models in promoting complete and sustainable motor development in the long term [27].

### 4.6. Practical Implications

From an applied perspective, the results are consistent with the potential value of multisport exposure in the early stages of motor development: the Multisport group, despite lower baseline coordination scores, showed greater improvements over time at the group level (Section 3.5). Given the non-randomized, 10-week design, these findings should be regarded as preliminary and hypothesis-generating rather than a basis for programmatic or policy recommendations.

It should be noted that this pattern was observed as a between-group comparison and was not tested as an individual-level association between baseline scores and change scores. In educational and sport settings, these findings may inform discussion of multilateral program design, pending confirmation in randomized or cluster-randomized studies. From a long-term developmental perspective, the improvement of motor coordination in childhood may favor greater participation in physical activity and contribute to more favorable health trajectories across the lifespan [1,4,43].

### 4.7. Limitations and Future Directions

This study presents several limitations. The design was quasi-experimental and non-randomized: children were assigned to groups based on the pre-existing program offered by their club, and unmeasured family- and club-level factors (e.g., parental sport involvement, socioeconomic context, coaching differences) may have contributed to the baseline differences observed between groups (Single-sport: 117.33 ± 15.82; Multisport: 106.15 ± 21.24). These baseline differences were addressed through ANCOVA and club-level mixed-effects models (Section 3.8 and Section 3.9), both of which confirmed the robustness of the between-group difference in change scores, although unmeasured confounding cannot be fully excluded. The 10-week observation window does not establish whether the observed adaptations persist over time. Internal training load was estimated using the RPE-C scale [21] rather than objective instruments (e.g., heart-rate monitors); RPE-C values were comparable between groups (MG: 7.6 ± 0.3; SG: 7.8 ± 0.2), but this measure does not capture training volume, density, or task complexity. Additional limitations include: the use of raw KTK scores rather than the age- and sex-adjusted Motor Quotient, limiting comparability with normative-based studies; the absence of formal inter-rater reliability indices for KTK administration; unassessed maturational status, which may have contributed to individual differences despite similar chronological age between groups; a relatively small number of females in the Single-sport group (*n* = 12), limiting power for sex × group interactions; and unassessed family-level factors such as physical activity habits. Future studies with larger, more balanced samples, cluster-randomized designs, and combined maturational and family-context data would help address these aspects more directly.

## 5. Conclusions

In conclusion, both practice models were associated with improvements in motor coordination over the 10-week observation period. Multisport practice was associated with a significantly greater magnitude of change, as indicated by both the analysis of change scores and the significant Time × Group interaction, a finding that remained robust after adjusting for baseline differences and club-level clustering (Section 3.8 and Section 3.9). Comparable perceived exertion between groups, assessed via the RPE-C scale [21], suggests that this difference is not attributable to exercise intensity. Given the non-randomized design and short observation window, these findings should be regarded as preliminary evidence that diversified, play-based motor experiences in early childhood merit further investigation in longer, randomized or cluster-randomized studies, rather than as a basis for immediate practice or policy recommendations.

## Figures and Tables

**Table 1:** Anthropometric characteristics of participants (mean ± SD).

Variable	Single-Sport	Multisport	*p*-Value
Age (years)	6.13 ± 0.53	6.28 ± 0.57	0.095
Body height (cm)	118.11 ± 3.70	121.33 ± 8.50	0.005
Body mass (kg)	20.89 ± 1.55	22.34 ± 3.32	0.002
BMI (kg/m^2^)	14.98 ± 0.69	14.95 ± 0.68	0.766

Note: Values are expressed as mean ± standard deviation. *p*-values refer to independent-samples *t*-tests comparing groups at baseline. Baseline differences in body height and body mass were addressed as potential confounding factors in the sensitivity analysis (Section 3.8).

**Table 2:** Comparison of training program characteristics.

Characteristic	Multisport Group	Single-Sport Group (Soccer)
Weekly frequency	2 sessions/week	2 sessions/week
Session duration	60 min	60 min
Program duration	Approximately 8 months	Approximately 8 months
Mean session RPE (RPE-C scale)	7.6 ± 0.3	7.8 ± 0.2
Training focus	General motor development	Soccer-specific skills
Task diversity (qualitative)	High (multiple motor domains)	Moderate (soccer-specific tasks)
Main activities	Multilateral exercises, circuits, games	Technical drills, small-sided games
Target skills	Balance, locomotion, manipulation, rhythm	Soccer-specific coordination
Teaching approach	Play-based, exploratory	Play-based, sport-specific
Sport exposure	Multiple sports	Single sport
Progression model	Multicomponent	Sport-specific
Coach qualifications	Certified sport science instructors (multilateral training)	Certified soccer coaches (national federation license)
Fidelity monitoring	Weekly session checklist completed by lead instructor	Weekly session checklist completed by lead coach

Note: Motor variability reflects the qualitative design and intended structure of the two programs as implemented by the respective sports clubs. RPE was assessed using the RPE-C scale [21], administered individually at the end of each training session.

**Table 3:** Descriptive statistics of KTK subtests and total RAW SCORE (mean ± SD).

Variable	Multisport T0	Multisport T1	Single-Sport T0	Single-Sport T1
WB (steps)	29.28 ± 7.59	33.35 ± 6.48	35.38 ± 6.37	37.42 ± 5.71
MS (moves)	26.75 ± 6.30	29.99 ± 6.62	28.27 ± 4.05	31.53 ± 3.98
HH (points)	18.20 ± 7.09	22.46 ± 7.57	22.08 ± 5.81	25.83 ± 6.36
JS (jumps)	31.92 ± 8.06	37.04 ± 8.69	31.61 ± 5.55	34.83 ± 5.76
RAW SCORE	106.15 ± 21.24	122.84 ± 23.17	117.33 ± 15.82	129.61 ± 15.96

**Table 4:** Within-group comparisons from T0 to T1.

Variable	Multisport *p*-Value	Multisport Cohen’s d	Single-Sport *p*-Value	Single-Sport Cohen’s d
WB	<0.001	0.58	<0.001	0.34
MS	<0.001	0.50	<0.001	0.81
HH	<0.001	0.58	<0.001	0.62
JS	<0.001	0.61	<0.001	0.57
RAW SCORE	<0.001	0.75	<0.001	0.77

**Table 5:** Between-group comparisons at T0 and T1.

Variable	T0 *p*-Value	T0 Cohen’s d	T1 *p*-Value	T1 Cohen’s d
WB	<0.001	−0.86	<0.001	−0.66
MS	0.097	−0.28	0.103	−0.28
HH	0.001	−0.59	0.005	−0.48
JS	0.791	0.04	0.083	0.29
RAW SCORE	<0.001	−0.59	0.049	−0.33

**Table 6:** Change scores (Δ = T1 − T0).

Variable	Multisport Δ	Single-Sport Δ	95% CI of Difference	*p*-Value	Cohen’s d
WB	4.08 ± 2.40	2.05 ± 1.53	(1.38, 2.68)	<0.001	0.99
MS	3.24 ± 2.38	3.27 ± 1.29	(−0.64, 0.59)	0.940	−0.01
HH	4.25 ± 2.93	3.75 ± 1.76	(−0.27, 1.28)	0.230	0.20
JS	5.11 ± 2.48	3.22 ± 1.76	(1.20, 2.59)	<0.001	0.87
RAW SCORE	16.68 ± 6.14	12.28 ± 4.08	(2.72, 6.09)	<0.001	0.83

Note: Δ = T1 − T0; CI = confidence interval.

**Table 7:** Results of the two-way repeated measures ANOVA (RAW SCORE).

Effect	F (df1, df2)	*p*-Value	η^2^p
Time	F(1, 141) = 1092.31	<0.001	0.886
Group	F(1, 141) = 7.51	0.007	0.051
Time × Group	F(1, 141) = 24.18	<0.001	0.146

Note: η^2^p = partial eta squared.

## Data Availability

The data presented in this study are available on request from the corresponding author.

## Supplementary Materials

The following supporting information can be downloaded at: https://www.mdpi.com/article/10.3390/jfmk11030295/s1.
