# Supplementary material for: Multisport vs. Single-Sport Training and Motor Coordination in Children: A Quasi-Experimental Pre–Post Comparison
Source: J Funct Morphol Kinesiol. 2026 Jul 27;11(3):295. doi: 10.3390/jfmk11030295 (PMC13510616; doi:10.3390/jfmk11030295)
Supplement: Supplementary file 1 [file jfmk-11-00295-s001.zip › jfmk-4414179-supplementary.pdf]

Supplementary Table S1

Benjamini–Hochberg false discovery rate (FDR)-corrected q-values for all 25 statistical comparisons across the four KTK subtests and the total RAW SCORE, referenced in Section 2.5 of the main manuscript.

Comparison	KTK Outcome	t	p-value	q-value (FDR)	Significant (q < 0.05)
Within-group, Multisport (T0 vs T1)	Walking Backward (WB)	15.12	<0.001	<0.001	Yes
Within-group, Single-sport (T0 vs T1)	Walking Backward (WB)	10.73	<0.001	<0.001	Yes
Within-group, Multisport (T0 vs T1)	Hopping for Height (HH)	8.11	<0.001	<0.001	Yes
Within-group, Single-sport (T0 vs T1)	Hopping for Height (HH)	17.01	<0.001	<0.001	Yes
Within-group, Multisport (T0 vs T1)	Jumping Sideways (JS)	18.36	<0.001	<0.001	Yes
Within-group, Single-sport (T0 vs T1)	Jumping Sideways (JS)	14.64	<0.001	<0.001	Yes
Within-group, Multisport (T0 vs T1)	Moving Sideways (MS)	12.12	<0.001	<0.001	Yes
Within-group, Single-sport (T0 vs T1)	Moving Sideways (MS)	20.29	<0.001	<0.001	Yes
Within-group, Multisport (T0 vs T1)	RAW SCORE (total)	19.68	<0.001	<0.001	Yes
Within-group, Single-sport (T0 vs T1)	RAW SCORE (total)	24.57	<0.001	<0.001	Yes
Between-group, T0	Walking Backward (WB)	-5.13	<0.001	<0.001	Yes
Between-group, T1	Walking Backward (WB)	-3.93	<0.001	<0.001	Yes
Between-group, T0	Hopping for Height (HH)	-3.52	<0.001	<0.001	Yes
Between-group, T1	Hopping for Height (HH)	-2.36	0.020	0.027	Yes
Between-group, T0	Jumping Sideways (JS)	0.27	0.791	0.824	No
Between-group, T1	Jumping Sideways (JS)	1.75	0.083	0.104	No
Between-group, T0	Moving Sideways (MS)	-1.67	0.097	0.116	No
Between-group, T1	Moving Sideways (MS)	-1.64	0.103	0.117	No
Between-group, T0	RAW SCORE (total)	-3.45	<0.001	0.001	Yes
Between-group, T1	RAW SCORE (total)	-1.75	0.083	0.104	No
Between-group, Δ (T1−T0)	Walking Backward (WB)	5.88	<0.001	<0.001	Yes
Between-group, Δ (T1−T0)	Hopping for Height (HH)	1.23	0.221	0.240	No
Between-group, Δ (T1−T0)	Jumping Sideways (JS)	5.16	<0.001	<0.001	Yes
Between-group, Δ (T1−T0)	Moving Sideways (MS)	-0.08	0.940	0.940	No
Between-group, Δ (T1−T0)	RAW SCORE (total)	4.62	<0.001	<0.001	Yes

Note: Within-group comparisons are paired t-tests (T0 vs. T1) within the Multisport (MG) and Single-sport (SG) groups. Between-group comparisons are independent-samples t-tests at T0, at T1, and on change scores (Δ = T1 − T0). q-values were calculated using the Benjamini–Hochberg procedure across all 25 comparisons simultaneously. WB = Walking Backward; HH = Hopping for Height; JS = Jumping Sideways; MS = Moving Sideways.
